# August

**Published:** 1890-08-09

**Authors:** 


### August.

August was formerly the sixth month of the year according to the pre-Julian Roman calendar, and was then termed *sextilis*. It received the name of August in honour of the Emperor Augustus, because it was the period of the year when the greatest good luck happened to him. As July contained thirty-one days, and as July was named after a former Emperor, it was thought necessary to add another day to August (which had previously only thirty days), so that Augustus might not be inferior to Julius! August is usually characterised by fine weather, and the rich glow of summer is still present. Forlong, in his "Rivers of Life," says: "Now rest, ease, or *vacuna*, is all that need engage man's attention, for Nature herself exhausted pauses, days and nights are again equal, and peace and plenty are diffused throughout her realms." This, however, relates more especially to the East, for in our northern and westerly clime man cannot yet rest from his labours, for the remnant of the hay has to be gathered, and the rapidly-turning colour of the cornfields foretells much labour ere the different crops may be garnered. For the first twenty days or so of August the mean average temperature, 62 deg., does not much differ from that of July, but towards the end of the month the temperature falls a degree or two, the average mean temperature of the last day of August being as low as 59. But although the days are often hot, the mornings and evenings possess a crispness which was not characteristic of the previous month. Still it is summer, and open country or the seaside is preferable to the crowded town. As in July, we may expect occasional thunderstorms, the accompanying rain being very serviceable in washing away the dirt and débris which will collect in hot, dry summer days. So London is still comparatively empty, all who can being still engaged in holiday making, either at home or abroad. The 1st of August is the gule or Lammas day, regarding the origin of which there has been considerable controversy. Some suppose it to have been derived from the Latin "gula," throat; this being the period at which the daughter of the Tribune Quirinus was cured of some disorder of the throat by kissing the chains of St. Peter. Lammas, however, has a different meaning, and is supposed to be a corruption of La-ith-mas, the ancient Druidical ceremony of sacrifice of the fruits of the soil. On August the 5th, 1738, a young gentleman advertised in the *London Chronicle* for a young lady who must have noticed him at Vauxhall, because he wore a blue and gold laced hat. It is added that his "intentions were strictly honourable." Vauxhall is a memory of the past, and chimney-pots have displaced blue and gold hats, but the "Agony Column" still survives! In August "shooting stars shine through heaven on nights between nine and eleven." St. Lawrence's tears they were called, because they were supposed to fall in showers about the time the saint was martyred. There was a popular idea that they brought ill-luck to any one who looked at them. When looking for shooting stars in August, we are quite as likely to see "the bat with giddy wing making his round of shed and tree," for it is at this time bats most do fly. Bats were also once regarded as prognosticating evil. August the 15th is the day of the Assumption of the Blessed Virgin. "The sun most glorious orb that wert a worship, 'ere the history of its making were revealed," has now almost done its work. August 16th, or St. Roche's Day, was long celebrated as the general harvest home, when—

> "The ears are filled, the fields are white,
> The constant harvest moon is bright."

But our harvests are now later than they appear to have been in bygone years. Once upon a time, St. Roche became sick, but by the aid of abstinence he recovered. Butler thinks that the expression "As sound as a roach," may have thus originated, for the fish of that name is not a particularly sound one. St. Roche was also regarded as the Saviour from Plague. Now "plague, pestilence, and famine," are closely allied. Plague is always most prevalent and destructive among the poor and destitute. Hence, probably, St. Roche, the harvest saint, came to be regarded as the saviour from plague, which diminished when the people had more food. At this time, in ancient Rome, it was customary to implore blessings on the fruits which had been garnered. August 24th, or St. Bartholomew's Day, is thus mentioned—

> "All the tears that St. Swithin can cry,
> St. Bartholomew's dusty mantle wipes dry."

Swallows are now preparing for their departure to the far south-west. The emigration of birds is a subject on which much has been written, and regarding which little is known. We confess our ignorance when we attribute it to instinct! Cicero said that between man and the lower animals there is the great distinction of the reasoning power of the former, by which he joins the future to the present, foreseeing and preparing for his path in life. But the reason of man does not enable him to accomplish what the instinct of animals enables them to surmount. Towards the end of August our grandmothers used to compound "blackberry jam," which a writer, signing "I. J. T." in 1826, designated "angels' food." There is, indeed, no accounting for tastes. Neither blackberries nor blackberry jam are much esteemed in this fastidious age. We have, however, sometimes thought that the blackberry has not been quite fairly treated. So far as we are aware it has never been submitted to high cultivation. It is quite possible that a fruit, more worthy of the designation "angels' food," might be developed from the wild blackberry. It is also in August that the glow-worm is found in greatest abundance. Unfortunately, we have no flying glow-worms in this country. It is related that some ancient ladies seeing fire-flies for the first time at some place abroad, caused the windows of their lodgings to be shut, under the idea that these shining flies were the souls of their deceased husbands! This seems to show, that in their case marriage had proved a failure! It is about the same time that "earwigs" most abound. There is a record that some years back these insects appeared in the neighbourhood of Stroud in tens of thousands. The once popular delusion that if one of these insects got into the ear madness followed, is now totally discredited. August 29th is esteemed as the anniversary of the beheading of St. John the Baptist, at the request of the beautiful Salome, the daughter of Herodias by her former husband. A writer in a London paper published about one hundred years ago said that this was the time when epidemical disorders of the bowels most prevail, and which are ascribed to eating fruits. The remark, as regards time, still holds good, but scientists now ascribe bowel complaints to microbes. Another old writer gave a prescription for the cure of bowel complaints: Confection of catechu, two drachms; syrup of white poppies, half-an-ounce; cinnamon water, four ounces; one ounce for a dose. This, he said, is preferable to the spirituous and narcotic preparations usually prescribed—apparently forgetting that syrup of white poppies is a narcotic. The following is not a bad illustration of the social changes which have taken place. In an old book it is stated that August may be considered the first of the sporting months. The legal shooting of red game, or grouse, commences on the 12th, and of black game, or heath-fowl, on the 20th. "But as these birds chiefly frequent the moorlands in Scotland and the North of England, they seldom arrive as presents in

London until the cold weather admits of their transmission to so great a distance in a state fit for the table." But we now live in other times, and would not be content with the old order of things. We now obtain our grouse by rail in London, long before they are fit for the table. For in our opinion, a grouse is none the worse for being hung a few days before it is eaten. Everyone, however, cannot regale on grouse, for grouse are expensive in these days when so many want the birds. Someone said that if truffles were a necessity of life, there would be much more misery than there is. The observation applies to grouse. In the "Gulistan" of the old Persian poet Saadi, is the following couplet: "Eating is for living and praise of God; you think that living is for eating." Let those who cannot get grouse remember this, and be thankful; even if they have no cook who "with art increases physicians' fees, and serves up death in soups and fricassees," for a very considerable proportion of the ailments afflicting humanity originate from too much indulgence in rich food.

